# Safety and Effectiveness of Subcutaneous Immunotherapy with a Glutaraldehyde-Polymerized Mite Allergen Extract in Adults and Children with Allergic Rhinitis with or Without Asthma Due to *Dermatophagoides*

**DOI:** 10.3390/diseases14020037

**Published:** 2026-01-23

**Authors:** Olalla Verdeguer Segarra, Zulay Almeida Sánchez, Silvia Quarta, Emilio Funes Vera, Óscar M. González Jiménez, Guacimara Hernández Santana, Leticia Herrero Lifona, Paula López-González, Montserrat Martínez-Gomariz, Beatriz López-Cauce, Aída Gómez-Cardenosa

**Affiliations:** 1 Allergy Unit, Hospital Francesc de Borja, 46702 Valencia, Spain; olalla.verdeguer@gmail.com; 2 Allergy Unit, Hospital Universitario de La Palma, 38713 Santa Cruz de Tenerife, Spain; zulay85@hotmail.com; 3 Allergology Service, Hospital Universitari Sagrat Cor, 08029 Barcelona, Spain; sil.quarta@gmail.com; 4 Allergology Unit, Hospital Universitario Rafael Mendez, 30817 Murcia, Spain; efunesvera@gmail.com; 5 Allergy Unit, Hospital Quirón, 08029 Barcelona, Spain; om.gonzalezjimenez@gmail.com; 6 Service of Allergy, Hospital Universitario Nuestra Señora de Candelaria, 38010 Santa Cruz de Tenerife, Spain; guacim1@hotmail.com; 7 Allergy Department, Hospital Quironsalud Málaga, 29004 Málaga, Spain; 8 Allergy Department, Infanta Leonor University Hospital, 28031 Madrid, Spain; paula.lopez@salud.madrid.org; 9 Research and Development Department, Diater Laboratories, 28919 Madrid, Spain; m.martinez@diater.com; 10 Medical Affairs Department, Diater Laboratories, 28919 Madrid, Spain; b.lopez@diater.com

**Keywords:** allergen immunotherapy, allergic rhinitis, allergic asthma, allergic conjunctivitis, *Dermatophagoides farinae*, *Dermatophagoides pteronyssinus*, glutaraldehyde-polymerized, house dust mites, maximum allergen extract concentrations, real-world data

## Abstract

Background/Objectives: The aim of this study was to evaluate the tolerability and effectiveness of subcutaneous immunotherapy (SCIT) in allergic adults and children treated with a polymerized-glutaraldehyde undiluted mixture of house dust mites (HDMs) under routine clinical practice. Methods: This was an observational, ambispective, controlled, real-world, multicenter study including patients ≥ 5 years with allergic rhinitis (AR), due to *Dermatophagoides* sensitization. Patients who started AIT with a *D. pteronyssinus*/*D. farinae* extract and those who continued symptomatic treatment were included in the treatment (DP&DF) and untreated (UT) groups, respectively. We evaluated adverse reactions (ARs) and changes in effectiveness variables through changes in symptoms, disease control, medication use, and patient- and investigator-reported outcomes. Results: We included 130 patients in the DP&DF group, and 90 (69.2%) adults, 23 adolescents (17.7%), 17 (13.1%) children, and 94 patients in the UT group. Patients received treatment for a mean (SD) of 9.01 (3.1) months at the time of evaluation. Seven (5.4%) patients, all adults, reported eight ARs, five local and three systemic (mean rate of 0.62 ARs per 100 injections); all recovered, and epinephrine was not required. The proportion of patients reporting no rhinitis symptoms at follow-up significantly increased (+13.6%; *p* < 0.001). Rhinitis frequency, intensity, and control significantly improved overall and in specific age groups. Similarly, the proportion of patients reporting no asthma symptoms at follow-up significantly increased (+29.0%; *p* < 0.001). The use of all symptomatic medications significantly decreased, while the UT group showed no significant changes, except for worsened asthma classification and control in specific age groups. Both investigators and patients perceived a marked improvement in symptoms and medication use, with high satisfaction scores reported on the visual analogue scale. Conclusions: A subcutaneous allergen extract with a mixture of HDMs is safe and effective for allergic rhinitis and asthma in adults and children in the real-world setting.

## 1. Introduction

Allergic rhinitis (AR) is considered a highly prevalent condition worldwide, with a median prevalence of 18.1%, despite considerable variability across regions and studies [1,2]. AR is frequently associated with asthma, and both are considered interconnected airway IgE-mediated inflammatory conditions [3]. Additionally, AR is frequently associated with allergic conjunctivitis [3,4,5]. These chronic allergic conditions impact patients’ social life, quality of life, and school and work performance, resulting in a significant burden on healthcare systems and society due to direct and indirect costs [6].

House dust mites (HDMs) are an important source of perennial aeroallergens associated with allergic rhinitis, asthma, and conjunctivitis in sensitized patients [7]. HDMs are common in indoor environments (i.e., at homes) worldwide, causing patients’ exposure year-round, and are the most frequent cause of perennial allergic respiratory disease, constituting a major public health problem [7,8,9]. In Spain, HDM allergy is the second most common cause of respiratory allergy after pollens [10]. *D. pteronyssinus* and *D. farinae* are the most common HDM species and the source of potent allergens with different mechanisms of action on the immune system [7,9]. Importantly, sensitization to perennial allergens such as HDMs in children determines the course of respiratory allergic disease [9,11].

Allergen immunotherapy (AIT) involves the administration of the allergen at controlled doses to induce immune tolerance. It is the only treatment that can control symptoms while modifying the disease course, providing a more permanent solution than conventional pharmacological treatment [12,13,14]. Clinical trials have proven its efficacy and safety in treating rhinitis and asthma, which have been further confirmed in real-world settings [15,16,17,18,19]. Allergen extracts polymerized with glutaraldehyde have reduced allergenicity while maintaining immunogenicity and have shown an improved safety profile compared to native extracts [20]. Compared with native allergen extracts, polymerized formulations are associated with a lower risk of systemic reactions, allowing the administration of higher allergen doses with improved tolerability [20]. In addition, while both subcutaneous and sublingual immunotherapy (SCIT and SLIT) have demonstrated efficacy and safety in respiratory allergy, SCIT allows greater flexibility in dosing and composition of allergen mixtures and remains a widely used approach in routine clinical practice, particularly in patients with moderate to severe disease or polysensitization [12,13,14]. Given that patients are frequently sensitized to multiple HDM species, AIT with allergen mixtures may offer advantages to these patients [10]. However, the effects of AIT are dose-dependent, and mixtures containing higher allergen doses are necessary to achieve the desired clinical benefit despite the potentially increased risk of adverse reactions [21]. In routine clinical practice, allergen mixtures are frequently diluted to reduce the risk of adverse reactions; however, dilution may result in suboptimal allergen doses and reduced clinical effectiveness, given the dose-dependent nature of allergen immunotherapy [21]. The use of undiluted polymerized allergen mixtures may allow the administration of higher cumulative allergen doses while maintaining an acceptable safety profile, particularly when cluster up-dosing schedules are applied under medical supervision. This approach aims to optimize the balance between tolerability and effectiveness in patients requiring immunotherapy with multiple allergens, reflecting real-world treatment strategies rather than idealized trial conditions.

This observational, ambispective study aimed to evaluate the tolerability of subcutaneous immunotherapy (SCIT) with an undiluted mixture of *D. pteronyssinus* and *D. farinae* allergen extract polymerized with glutaraldehyde in patients with rhinitis, with or without asthma, in routine clinical practice. Secondary objectives were to assess the effectiveness of the AIT and the relationships between tolerability and demographic, clinical, and treatment variables.

## 2. Materials and Methods

### 2.1. Study Design and Population

This was an observational, ambispective (retrospective and cross-sectional), controlled, real-world multicenter study including patients ≥ 5 years old with allergic rhinitis, with or without controlled asthma, due to sensitization to *D. pteronyssinus* and/or *D. farinae*, *O. europaea* and/or wild grasses, or *D. pteronyssinus* and/or *B. tropicalis*, according to skin prick tests and/or specific IgE determinations. We carried out this study at 30 participating centers.

Patients initiated subcutaneous immunotherapy between September 2021 and June 2022. Data were retrospectively and cross-sectionally collected from medical records between October 2022 and March 2023, including information from baseline (before AIT initiation) and the follow-up visit according to routine clinical practice.

Some patients presented additional sensitization to pollen allergens; however, pollen sensitization was not considered clinically relevant at the time of inclusion and was not the target of allergen immunotherapy in this subanalysis.

This report is a subanalysis of patients who started AIT with an undiluted *D. pteronyssinus*/*D. farinae* extract, according to the routine clinical practice between September 2021 and June 2022. All patients who started this AIT were included in the treatment group (DP&DF) regardless of the total number of AIT doses received. Patients sensitized to the study allergens (*D. pteronyssinus* and/or *D. farinae*, *O. europaea* and/or wild grasses, or *D. pteronyssinus* and/or *B. tropicalis*) who did not start AIT during the same period and continued with their conventional symptomatic treatment were included in the control group (Untreated, UT). Patients with no available medical history and who had received AIT with *D. pteronyssinus* and/or *D farinae* extracts in the previous five years were excluded. Data were collected from medical records between October 2022 and March 2023 and included data before starting the AIT and after starting the AIT, the latter visit corresponding to the follow-up visit according to routine clinical practice. Moreover, patients and investigators completed specific questionnaires to evaluate the AIT, which were administered during the recruitment visit to patients and after patients’ inclusion in the study to the investigators (cross-sectional data).

The study was conducted according to the principles of the Helsinki Declaration (Fortaleza, Brazil) and the local data protection regulations (Spanish Organic Law 15/1999, of 13 December). All included patients and the legal representatives of patients < 18 years old signed a written informed consent before any information was recorded. The Independent Ethics Committee (Comité de Ética para la Investigación con medicamentos, CEIm) of Getafe University Hospital (Madrid, Spain) approved the study protocol.

### 2.2. Study Medication

Patients received AIT with the undiluted mixture of *D. pteronyssinus* and *D. farinae allergen* extract (*D. pteronyssinus*/*D. farinae* extract) polymerized with glutaraldehyde in an aqueous suspension for subcutaneous administration (Polymerized 100, Diater Laboratories, Madrid, Spain).

Each vial of the undiluted extract (vial 3) contained 0.3 HEPD/mL of *D. farinae* and 0.3 HEPD/mL of *D. pteronyssinus*, according to the manufacturer’s specifications. During the maintenance phase, patients received a monthly dose of 0.5 mL of vial 3, corresponding to an administered dose of 0.15 HEPD of *D. farinae* and 0.15 HEPD of *D. pteronyssinus* per injection.

The AIT was administered in two phases: a build-up phase, consisting of increasing doses until reaching the 0.5 mL maintenance dose, and a maintenance phase, consisting of monthly 0.5 mL injections for at least three years. In the build-up phase, the dose administrations may follow two possible schedules: a cluster schedule consisting of a 0.2 mL and a 0.3 mL dose on the same day at a 30 min interval and a conventional two-week schedule consisting of a single dose of 0.2 mL at day 1 and 0.5 mL at day 8.

### 2.3. Objectives and Variables

The primary objective, which was tolerability, was assessed as adverse reactions (ARs), including systemic reactions (SRs) and local reactions (LRs), per injection administered (ARs/100 injections or percentage of injections with ARs). We also considered the time of onset (immediate/late). Immediate reactions were those occurring within 30 min of injection, and late reactions were those occurring >30 min after injection. SRs were classified according to the World Allergy Organization (WAO) criteria [22]. Secondary tolerability objectives were to evaluate the relationship between ARs and the administration phase, clinical manifestations of allergy (rhinitis vs. rhinoconjunctivitis, asthma vs. no asthma), and demographic variables (i.e., age and sex).

We assessed effectiveness (secondary objective) as changes in allergy symptoms (frequency, intensity/severity, and control), including rhinitis and asthma, and medication use from before starting AIT until the routine clinical visit after starting AIT. Rhinitis was classified according to the Allergic Rhinitis and its Impact on Asthma (ARIA) guidelines based on frequency, intensity, and control [23,24]. Asthma was classified according to the Guía Española para el Manejo del Asma 5.0 (GEMA) based on frequency and severity, treatment classification steps, and control [25]. The symptomatic medications included oral, nasal, and ocular antihistamines, oral, nasal, and inhaled corticosteroids, β2 inhaled agonists, and anti-leukotrienes, and their use was evaluated as yes and no. The evolution of symptoms and the use of symptomatic medication were evaluated in all patients in the DP&DF and the UT groups. These variables were additionally analyzed according to age groups, including children (5 to 11 years), adolescents (12 to 17 years), and adults (≥18 years), in patients in the DP&DF group.

In addition to standard clinical measures, investigators and patients provided their views on AIT effectiveness compared with the period before starting AIT using specific questionnaires. They rated perceived changes in three allergy symptoms (rhinitis, conjunctivitis, and asthma) and the overall use of symptomatic medication with three possible responses (less, same, and more). Moreover, patients and investigators rated global satisfaction with the AIT on a visual analog scale (VAS) ranging from 0 to 100, with 100 representing the highest satisfaction level.

Baseline variables considered included demographic characteristics (i.e., age and sex), characteristics of the allergic disease, including diagnoses, sensitization profile, according to skin prick test results, and specific IgE (sIgE) results (sIgE ≥ 0.35 KU/L is considered positive). The allergens included in skin prick tests were *O. europea,* wild grasses, *D. pteronyssinus*, *D. farinae*, and *B. tropicalis*; sIgE determinations included *O. europea*, wild grasses, *D. pteronyssinus*, *D. farinae*, *B. tropicalis*, and sIgE to the major allergens Ole e 1, Phl p 1, Phl p 5, Der p 1, Der p 2, and Der f 1, with variations among hospitals depending on clinical routine assessments. AIT treatment characteristics included time from AIT treatment initiation to data collection (months) and AIT schedule.

Data were collected retrospectively and prospectively from medical records using standardized data collection forms specifically designed for the study. Investigators from all participating centers received common instructions regarding variable definitions and data recording procedures. Prior to analysis, data completeness and internal consistency were reviewed through logical checks to identify implausible or inconsistent values, which were clarified with the corresponding center when necessary. Missing data for specific variables (e.g., skin prick test results or specific IgE determinations) were not imputed, and analyses were conducted based on available data. The number of patients included in each analysis is specified where applicable.

### 2.4. Statistical Analysis and Sample Size

Considering a 20% prevalence of AR in the Spanish population, 502 patients were deemed necessary to evaluate the primary objective with a 3.5% precision and a 95% confidence interval, and without the need to replace patients, given the retrospective nature of this study [26].

Categorical variables were described as frequencies and percentages, and quantitative variables as the mean and standard deviation (SD) or the median and the 25th (first quartile, Q1) and 75th (third quartile, Q3) percentiles (Q1, Q3 or interquartile range [IQR]). The relationships between the incidence of ARs and demographic and clinical variables were evaluated using the Chi-Square and unilateral Fisher tests with a 95% confidence level. Variables before and after AIT were compared using the McNemar test, the Chi-Square test, and Fisher’s one-sided statistic with a 95% confidence level. Investigators’ perceptions in the DP&DF and UT groups were compared using the Chi-Square test. Statistical significance was set at a two-tailed α < 0.05. All statistical analyses were performed using the statistical package SAS system v9.4 and subsequent versions.

## 3. Results

### 3.1. Characteristics of Study Patients and Treatments

We recruited 465 patients, of whom eight were not valid because they were discharged, and no data were recorded for any variables (n = 6) or primary variables (n = 2). Of the 457 valid patients, 94 did not receive AIT (UT group), and 363 received AIT: 218 (47.7%) with mixtures of *Olea europaea*/wild grasses extracts, 130 (28.45%) with mixtures of D. *pteronyssinus/D farinae* extracts (DP&DF group), and 15 (3.28%) with mixtures of *D. pteronyssinus/Bloomia tropicalis* extracts.

In the DP&DF group, the mean (SD) age was 28.6 (14.7) years, with equal representation of female (53.8%) and male (46.2%) patients. Over two-thirds of patients were adults (n = 90, 69.2%), and almost one-third were pediatric patients < 18 years (n = 40, 30.8%), including children (n = 17, 13.1%) and adolescents (n = 23, 17.7%) (Table 1). The distribution of patients according to age was similar in the UT group. In the DP&DF group, over half of the patients had asthma (53.1%) or conjunctivitis (58.5%).

Most patients had persistent (94.4%) and moderate (73.6%) rhinitis, with partial and bad control (41.6% and 53.6%) at similar proportions (Table 1). Asthma was persistent moderate in over half of adult patients (55.6%) and frequent episodic in almost half (47.6%) of pediatric patients. Asthma control was mostly partial and good/total in adults (75.6%) and pediatric patients (95.2%). Table S1 summarizes the diagnoses and the characteristics of rhinitis and asthma across the three age groups.

Regarding the sensitization profile, approximately half of the patients in the DP&DF group were sensitized to other allergens in addition to *D. pteronyssinus* and *D. farinae*, as determined by skin prick tests and sIgE determinations (54.6% and 46.9%, respectively). Still, sensitizations to other allergens were not considered clinically relevant according to the physicians’ criteria (Table S2).

Patients received treatment for a mean (SD) of 9.01 (3.1) months (range: 4.2–22.7) at the time of the follow-up visit, according to routine clinical practice, corresponding to at least three complete doses, and were followed up for 4.20 to 15.43 months. The most frequent AIT schedule was the cluster in 112 (86.2%) patients; 15 (11.5%) patients followed the conventional schedule, and three (2.3%) patients had an unspecified schedule.

### 3.2. Tolerability of AIT

Seven (5.4%) patients, all adults, reported a total of eight ARs, of which five (62.5%) were LRs, and four (50.0%) were immediate, and occurred mainly in the up-dosing phase (62.5%) (Table 2). Study patients received a total of 1301 injections, resulting in a mean overall rate of 0.62 ARs per 100 injections (0.62%) and an AR rate in adults of 0.89%.

Of the five LRs, two (80.0%) occurred in the up-dosing phase. LRs were mostly immediate (60.0%) and mild (60.0%); no serious LRs occurred, and all patients recovered without additional medical treatment. Two of the three SRs (66.7%) occurred in the maintenance phase. Two (66.7%) SRs were Grade II (bronchospasm and unreported) and were immediate, and one of them was severe (not specified). All the SRs were resolved without epinephrine treatment, no additional observation, or visit to emergency services (Table 2).

The occurrence of adverse reactions showed no relationships with demographic variables, including age group and sex, clinical variables, including rhinitis and asthma diagnosis, and treatment variables, including the up-dosing schedule (Table 3).

### 3.3. Evolution of Rhinitis Symptoms

**Figure 1:**
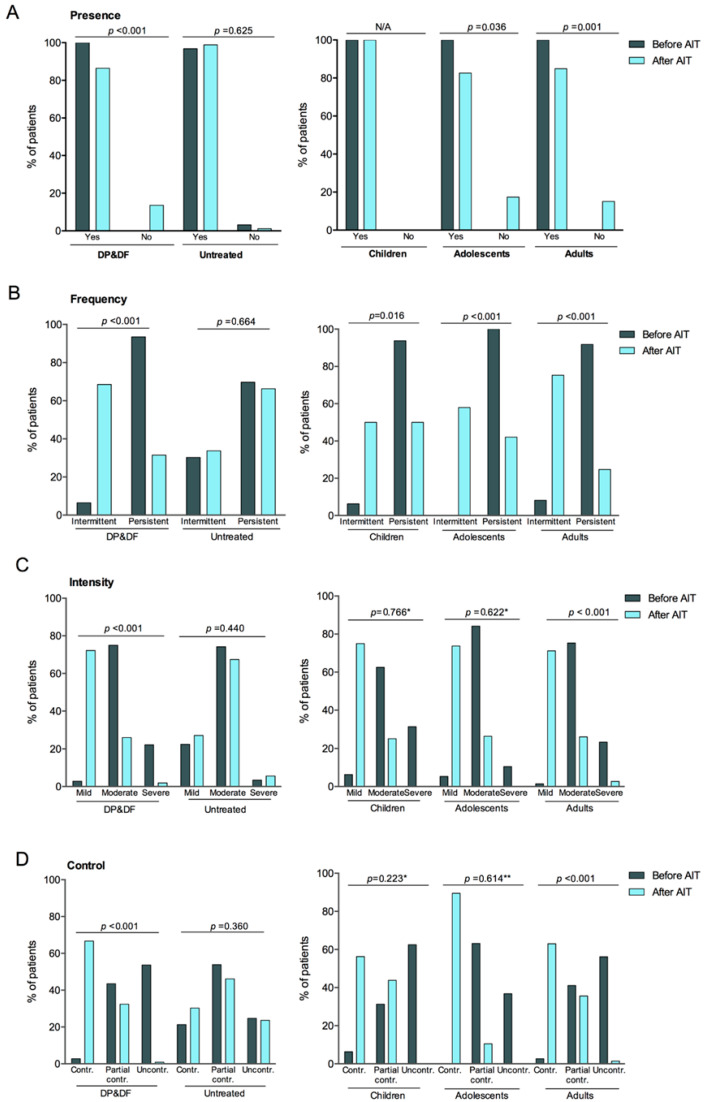
Rhinitis symptoms before and after AIT, including presence (**A**), frequency (**B**), Severity (**C**), and Control (**D**), based on the Allergic Rhinitis and its Impact on Asthma (ARIA) classification according to treatment (DP&DF vs. untreated) and, in treated patients, age group (children, adolescents, and adults). AIT, allergen immunotherapy. *p*-values comparing the distribution of patients before and after AIT were calculated using the McNemar test except for * *p*-value calculated using the Chi-square statistic with 95% confidence and ** *p*-value calculated using Fisher’s one-sided statistic.

In the DP&DF group, patients without rhinitis symptoms increased by +13.6 percentage points overall (*p* < 0.001), +17.4 points in adolescents (*p* = 0.036), and +15.1 points in adults (*p* = 0.001) after AIT but remained similar in children (Figure 1A). Rhinitis frequency significantly shifted from mostly persistent before AIT (93.5%) to mostly intermittent after AIT (68.5%), corresponding to −62.0% in patients with persistent rhinitis and +62.0% in patients with intermittent rhinitis (*p* < 0.001). Changes remained significant across age groups: persistent rhinitis decreased more in adults (−67.1 points) (*p* < 0.001), followed by adolescents (−57.9 points, *p* < 0.001) and children (−43.8 points, *p* = 0.016), indicating a favorable evolution of rhinitis symptoms after AIT regardless of age (Figure 1B).

Rhinitis intensity decreased in more severe categories after AIT and increased in less severe categories (*p* < 0.001). Adult patients showed a −20.6-percentage-point decrease in severe symptoms with a concomitant +69.8-percentage-point increase in mild symptoms (*p* < 0.001). Changes followed similar trends in children and adolescents but failed to reach statistical significance (*p* = 0.766 and *p* = 0.622, respectively) (Figure 1C). Likewise, controlled rhinitis significantly increased overall (+63.9 percentage points), while uncontrolled rhinitis decreased (−52.8 points) after AIT (*p* < 0.001). Changes remained significant in adults, with a +60.3-percentage-point increase in controlled rhinitis and a −54.8-percentage-point decrease in uncontrolled (*p* < 0.001), and followed similar trends in children and adolescents, without statistical significance (*p* = 0.223 and *p* = 0.614, respectively) (Figure 1D).

None of the rhinitis symptom measures showed significant changes in the UT group (Figure 1).

### 3.4. Evolution of Asthma Symptoms

**Figure 2:**
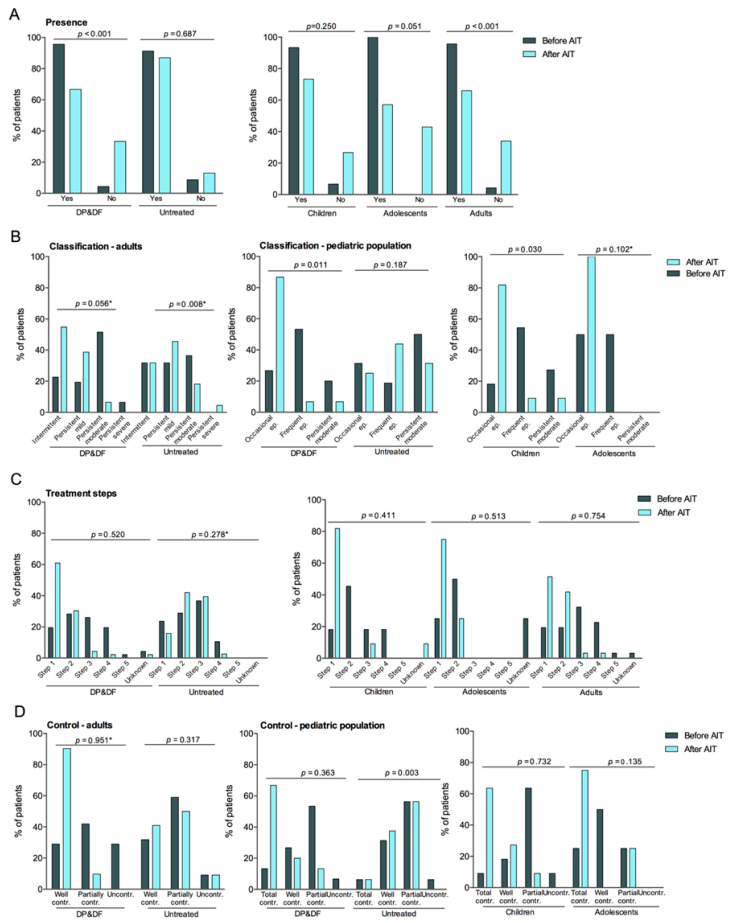
Asthma symptoms before and after AIT, including presence (**A**), classification based on GEMA (**B**), treatment steps (**C**), and Control (**D**), according to treatment (DP&DF vs. untreated) and, in treated patients, age group (children, adolescents, and adults). Asthma classification and control uses different categories in adults and pediatric populations and are represented in different graphs. AIT, allergen immunotherapy. *p*-values comparing the distribution of patients before and after AIT were calculated using the McNemar test except for * *p*-value calculated using the Chi-square statistic with a 95% confidence.

In the DP&DF group, patients without asthma symptoms significantly increased by +29.0 percentage points (*p* < 0.001) in the overall population after AIT. Changes were significant in adults, with a +29.7-point increase (*p* < 0.001), and in the pediatric population, with a +27.3-point increase (*p* = 0.031), whereas in children and adolescents, changes did not reach statistical significance (*p* = 0.250 and *p* = 0.051, respectively) (Figure 2A). Asthma classification significantly changed in the pediatric population, with a −13.3-point decrease in persistent moderate asthma and a +60.0-percentage point increase in occasional episodic asthma (*p* = 0.011). Changes followed a similar trend in adults but were not significant (*p* = 0.056) and were also similar in children and adolescents, reaching significance in children (*p* = 0.030) (Figure 2B).

Asthma treatment shifted towards lower steps in the overall population and according to age, although the changes were not significant (Figure 2C). Likewise, asthma control showed a trend toward improved control in adults and the pediatric population, with +61.3 and +53.4 percentage-point increases in well-controlled asthma and −29.0 and −6.7 percentage-point decreases in uncontrolled asthma, respectively, but did not reach statistical significance. Results were similar in children and adolescents, with a trend towards improved control, but changes did not reach statistical significance (Figure 2D).

Changes in asthma symptoms in untreated patients showed a trend towards worsening but were not significant, except for asthma classification in adults, with increased patients with persistent mild and persistent severe asthma (*p* = 0.008), and asthma control in the pediatric population, with increased uncontrolled asthma (*p* = 0.003), indicative of asthma symptoms worsening (Figure 2).

### 3.5. Evolution of Symptomatic Medication Use

**Figure 3:**
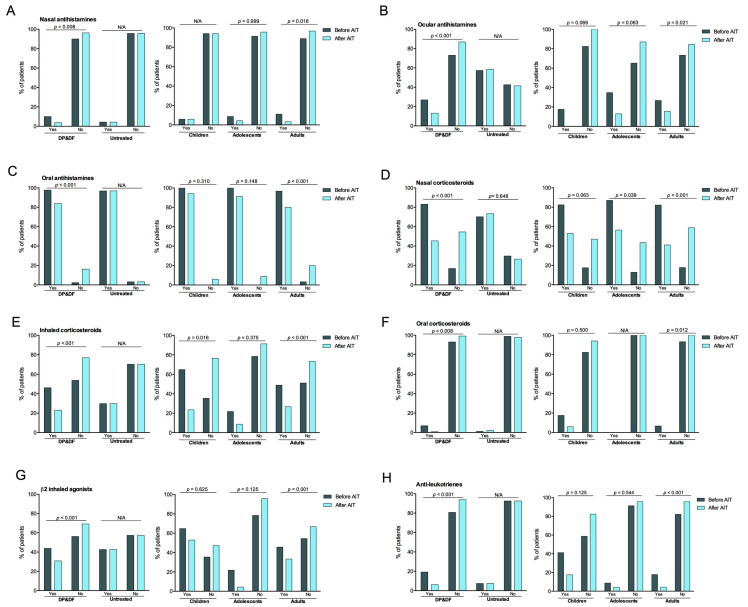
Use of rescue medication for rhinitis, conjunctivitis, and asthma before and after AIT, including nasal antihistamines (**A**), ocular antihistamines (**B**), oral antihistamines (**C**), and nasal corticosteroids (**D**), inhaled corticosteroids (**E**), oral corticosteroids (**F**), β2 inhaled agonists (**G**), and anti-leukotrienes (**H**), according to treatment (DP&DF vs. untreated) and, in treated patients, age group (children, adolescents, and adults). AIT, allergen immunotherapy. *p*-values comparing the distributions of patients before and after AIT were calculated using McNemar’s test with 95% confidence.

In the DP&DF group, the use of medication significantly decreased after AIT, particularly nasal corticosteroids (−37.7 points, *p* < 0.001) and inhaled corticosteroids (−23.1 points, *p* < 0.001), followed by oral antihistamines (−13.9 points, *p* < 0.001), ocular antihistamines (−13.8 points, *p* < 0.001), β2 inhaled agonists (−13.0 points, *p* < 0.001), anti-leukotrienes (−13.0 points, *p* < 0.001), nasal antihistamines (−6.2 points, *p* = 0.008), and oral corticosteroids (−6.1 points, *p* = 0.008) (Figure 3). The decreases were consistent in the three age groups and were significant in adolescents (−30.5 points, *p* = 0.039) and adults (−41.1 points, *p* < 0.001) for nasal corticosteroids and in children (−41.2 points, *p* = 0.016) and adults (−22.2 points, *p* < 0.001) for inhaled corticosteroids. The decreases were significant in adults for the use of antihistamines (nasal: −41.2 points, *p* = 0.016, oral: −16.7 points, *p* < 0.001, ocular: −11.1 points, *p* = 0.021), oral corticosteroids (−6.7 points, *p* = 0.012), antileukotrienes (−13.4 points, *p* < 0.001), and β2 inhaled agonists (−12.3 points, *p* = 0.001) but not in children and adolescents: antihistamines (nasal: −0 points, oral: −5.9 points, ocular: −17.6 points,), oral corticosteroids (−11.7 points), antileukotrienes (−23.6 points), and β2 inhaled agonists (−11.8 points) in children and antihistamines (nasal: −4.3 points, oral: −8.7 points, ocular: −21.8 points,), oral corticosteroids (−0 points), antileukotrienes (−4.4 points), and β2 inhaled agonists (−17.4 points) in adolescents (Figure 3).

None of the medications significantly changed their use in the UT group (Figure 3).

### 3.6. Investigators’ and Patients’ Points of View

Investigators perceived that, after AIT, most patients in the DP&DF group had fewer rhinitis (85.4%), conjunctivitis (57.7%), and asthma symptoms (49.2%). These percentages were higher in the DP&DF group than in the UT group for all symptoms (rhinitis: 17.0%, conjunctivitis: 12.8%, asthma: 14.9%) (Figure S1). Accordingly, investigators perceived that most patients in the DP&DF group used fewer symptomatic medications (87.7%) compared to 14.9% in the UT group (Figure S1). The mean (SD) satisfaction VAS score was 82.11 (16.52) points, indicating that investigators were very satisfied with the overall AIT response, compared to the mean (SD) 56.25 (21.40) points in the UT group.

In line with the investigators’ point of view, adult patients in the DP&DF group reported less rhinitis (86.1%), conjunctivitis (86.8%), and asthma symptoms (88.9%) after AIT, compared to 20.0%, 11.7%, and 8.3% in the UT group, respectively (Figure S2). Similarly, among adolescents, 95.7% reported fewer rhinitis symptoms, 87.5% fewer conjunctivitis symptoms, and 85.7% fewer asthma symptoms, compared to 5%, 15%, and 30% in the UT group, respectively. Moreover, patients in the DP&DF group reported decreased medication use for rhinitis, conjunctivitis, and asthma symptoms at higher frequencies than untreated patients, regardless of age group.

## 4. Discussion

The results from this observational, controlled study showed that a SCIT mixture of *D. pteronyssinus*/*D. farinae* allergen extracts polymerized with glutaraldehyde at maximum concentrations was well tolerated in children, adolescents, and adults, with a low incidence of ARs, all mild or moderate and occurring just in adult patients, resulting in a very low AR rate (0.62 AR per 100 injections). The treatment was effective in improving the symptoms of AR and asthma, with significant improvements across all parameters in the entire study population (except asthma treatment steps), despite the substantial prevalence of polysensitized patients, and in some age groups, particularly adults and adolescents. Consistently, medication use decreased significantly, reflecting the favorable disease evolution. Investigators and patients perceived the treatment as effective and reported being satisfied with its outcomes.

From a mechanistic perspective, the clinical benefits observed with allergen immunotherapy are supported by well-established immunological pathways involved in allergic inflammation. Allergic rhinitis and asthma are characterized by a Th2-skewed immune response, with increased activity of allergen-specific IgE, eosinophils, mast cells, basophils, and type 2 cytokines such as interleukin (IL)-4, IL-5, and IL-13. Allergen immunotherapy has been shown to modulate these pathways by inducing regulatory T cells, increasing allergen-specific IgG4, and reducing Th2-driven inflammation, ultimately leading to improved immune tolerance and clinical symptom control, as previously described [27].

AIT is considered safe, although tolerability, particularly for SRs, remains a concern [28]. Previous studies reported AR rates ranging from 1.3% to 3.6% of injections in patients receiving SCIT for different allergens [29,30,31]. The incidence of ARs to SCIT with polymerized extracts is generally lower, with reported rates of 0.71% in adults, in line with this study’s 0.89% [32], while other studies have reported rates of 0.1–0.236% for LRs and 0.4–0.456% for SRs [33,34]. Remarkably, in our study, few ARs occurred (n = 8), and the rate of ARs (0.61%) remained similar to previous reports, despite mixing HDM extracts at maximum concentrations. Remarkably, none of the ARs occurred in the 40 children and adolescents included, unlike previous studies reporting rates of 1.6% and 0.4% for polymerized extracts [32,35].

To our knowledge, few studies have assessed SCIT with diluted mixtures of allergen extracts from different HDMs, all in the trial setting, showing an acceptable tolerability profile [36,37]. Even though the low rate of ARs in this real-world study precluded robust comparisons with other studies, the profile of ARs was overall similar to that observed in previous real-world studies assessing polymerized allergen extracts. These studies reported that SRs were more frequently delayed and occurred in the maintenance phase, whereas LRs were primarily immediate, as in this study [32]. In our study, we found no relationships between ARs and age, sex, rhinitis diagnosis, asthma diagnosis, or up-dosing schedule, which was mostly cluster, further supporting the safety of the AIT across different patient profiles and treatment schedules. Furthermore, despite asthma control being bad in 24.4% of adults and 4.8% of the pediatric population, only one severe SR occurred, which was resolved with no medication. Importantly, no ARs were reported among the pediatric population, which represented one-third of the total population, underscoring the safety of this AIT in pediatric patients, consistent with previous large, real-world studies that concluded AIT is safe in this population [37,38].

AIT resulted in improved rhinitis and asthma symptoms, with concomitant decreased medication use, indicating a favorable disease evolution, which was not observed in the control group during the same period. These results align with previous studies showing favorable outcomes in patients with respiratory allergy to HDM [39]. Similarly to rhinitis symptoms, the presence of asthma decreased significantly, and asthma classification changed significantly, indicating a favorable disease course. The analysis stratified by age showed a trend toward improved rhinitis symptoms across age groups, but, except for rhinitis frequency, changes lacked statistical significance in the pediatric population. Remarkably, asthma classification also improved in the pediatric population, with significant changes in children, as shown in previous real-world studies [40]. Overall, AIT resulted in improved rhinitis and asthma symptoms, although in some age groups the changes did not reach statistical significance, likely due to the underrepresentation of the pediatric population, particularly children. The lack of statistical significance observed in some subgroup analyses, particularly in the pediatric population, is likely multifactorial. In addition to the limited sample size of children and adolescents, the variability in treatment duration and cumulative allergen exposure at the time of evaluation may have reduced the ability to detect statistically significant differences, despite consistent trends toward clinical improvement. Moreover, the real-world design of the study, with visits aligned to routine clinical practice rather than fixed time points, may have further contributed to variability in outcome assessment.

From a clinical perspective, these results nevertheless provide relevant guidance for routine practice. The favorable safety and tolerability profile observed across age groups, including children and adolescents, supports the use of the administration schedules applied in this study, including cluster schedules, under real-world conditions. Although this study was not designed to establish specific dosage adjustments for pediatric patients, the absence of severe adverse reactions and the consistent improvement trends suggest that current SCIT dosing strategies with polymerized HDM extracts are feasible and safe in pediatric populations when applied according to routine clinical practice.

Therefore, further studies in larger pediatric populations with longer and more homogeneous follow-up may yield statistically significant changes in rhinitis and asthma symptoms in children and adolescents. Moreover, the effectiveness of the AIT was evaluated after a mean treatment duration of 9 months (range: 4.2–22.7 months), which represents a relevant limitation of this study. It is well established that the clinical effectiveness of SCIT develops progressively over time and is closely related to the cumulative allergen dose. Therefore, the wide variability in treatment duration and cumulative exposure may have influenced the magnitude of the observed clinical improvements and may have led to an underestimation of the full therapeutic effect.

Consistent with the previous results, changes in rhinitis and asthma symptoms showed an improvement trend that did not significantly impact medication use in the pediatric population, likely due to insufficient sample size and follow-up. Remarkably, this formulation showed a favorable safety profile and was effective despite over half of the study population being sensitized to other allergens besides HDMs. The favorable safety profile of the AIT in the pediatric population and its potential to modify the allergic disease course and prevent asthma development, as shown in previous studies, warrant larger future studies assessing this AIT in this population [41,42].

The results of this study should be interpreted in the context of intrinsic limitations associated with its retrospective design, including missing data for some variables. The inclusion of untreated patients in this study as a control group aimed to reflect the natural evolution of the disease, which may not exactly match the sensitizing treatment allergen and kind of allergy (perennial vs. seasonal vs. both). Nevertheless, the results in the UT group reflect the outcomes of patients receiving conventional symptomatic treatment, suggesting that initiating AIT in the DP&DF group improved rhinitis and asthma symptoms. Moreover, the retrospective nature of this study precluded assessing standardized variables, such as the combined Symptoms and Medication Score (cSMS), as recommended to reduce heterogeneity and allow comparisons among studies [43]. Regarding the study size, it was calculated considering the analysis of all included patients, and the sample size in the DP&DF group was optimal to achieve sufficient statistical power for the global subanalysis of the AIT with HDM extract. However, the representation of the pediatric population, particularly children, was insufficient to obtain enough statistical power for the age-stratified analysis. Given the real-world nature of this study, with study visits matching those scheduled in the routine clinical practice, treatment duration and, consequently, the number of doses received at the time of evaluation were variable. Therefore, patients may have received the AIT for less than one year, likely resulting in an underestimation of its effects. Further studies with longer follow-up are needed to assess the effectiveness of the AIT in optimal conditions. Despite these limitations, this study captured the tolerability and effectiveness of this AIT in an unselected population of patients treated according to routine clinical practice, which included patients with polysensitization (i.e., HDMs + other allergens) and uncontrolled asthma.

Although some patients presented additional sensitization to other allergens, pollen sensitization was not considered clinically relevant at the time of inclusion and was not the target of immunotherapy in this subanalysis. Therefore, a formal comparison between monosensitized and polysensitized patients was not performed.

The results of this study provide valuable information supporting the excellent tolerability and effectiveness of AIT with a mixture of *D. pteronyssinus* and *D. farinae* extracts polymerized with glutaraldehyde at the maximum concentration for the treatment of allergic rhinitis and asthma.

## 5. Conclusions

AIT with a mixture of HDMs allergen extracts polymerized with glutaraldehyde at maximum concentrations resulted in improved allergic rhinitis and asthma symptoms and decreased use of medication in patients of all age groups with allergic rhinitis, with and without asthma, with a low incidence of ARs, no ARs in children and adolescents, and all patients continuing treatment, supporting the safety and effectiveness of this AIT strategy in the real-world setting.

## Figures and Tables

**Table 1:** Demographic and clinical characteristics of study patients according to treatment group. n (%).

	DP&DF n = 130	Untreated n = 94
**Demographic characteristics**		
**Age groups, n (%)**		
Children (5–11 years)	17 (13.1)	14 (14.9)
Adolescents (12–17 years)	23 (17.7)	20 (21.3)
Adults (≥18 years)	90 (69.2)	60 (63.8)
**Baseline allergic diagnosis, n (%)**		
Allergic rhinitis	125 (96.2)	93 (98.9)
Allergic conjunctivitis	76 (58.5)	70 (74.5)
Asthma	69 (53.1)	44 (46.8)
**Diagnoses combinations, n (%)**		
Rhinitis only	23 (17.7)	14 (14.9)
Rhinitis + conjunctivitis	38 (29.2)	36 (38.3)
Rhinitis + asthma	26 (20.0)	9 (9.6)
Rhinitis + conjunctivitis + asthma	38 (29.2)	34 (36.2)
Asthma only	5 (3.8)	1 (1.1)
Conjunctivitis	0 (0)	0 (0)
**Rhinitis characteristics (ARIA)**	n = 125	n = 90
**Frequency, n (%)**		
Intermittent	7 (5.6)	27 (30.0)
Persistent	118 (94.4)	63 (70.0)
**Severity, n (%)**		
Mild	3 (2.4)	20 (22.2)
Moderate	92 (73.6)	66 (73.3)
Severe	30 (24.0)	4 (4.4)
**Control, n (%)**		
Controlled	6 (4.8)	19 (21.1)
Partially controlled	52 (41.6)	48 (53.3)
Bad control	67 (53.6)	23 (25.6)
**Asthma characteristics (GEMA 5.0)**	n = 66	n = 42
Asthma treatment steps, n (%)		
Step 1	11 (16.7)	12 (28.6)
Step 2	21 (31.8)	12 (28.6)
Step 3	21 (31.8)	14 (33.3)
Step 4	10 (15.2)	4 (9.5)
Step 5	1 (1.5)	0 (0)
Step 6	0 (0)	0 (0)
Not available	2 (3.0)	0 (0)
**Asthma classification**		
Adults	n = 45	n = 25
Intermittent	10 (22.2)	8 (32.0)
Persistent Mild	8 (17.8)	9 (36.0)
Persistent Moderate	25 (55.6)	8 (32.0)
Persistent Severe	2 (4.4)	0 (0)
Pediatric patients	n = 21	n = 17
Occasional Episodic	7 (33.3)	6 (35.3)
Frequent Episodic	10 (47.6)	3 (17.6)
Persistent Moderate	4 (19.0)	8 (47.1)
Persistent Severe	0 (0)	0 (0)
**Asthma control**		
Adults	n = 45	n = 25
Well controlled	12 (26.7)	10 (40.0)
Partially controlled	22 (48.9)	13 (52.0)
Bad controlled	11 (24.4)	2 (8.0)
Pediatric patients	n = 21	n = 17
Total	2 (9.5)	1 (5.9)
Good	8 (38.1)	6 (35.3)
Partial	10 (47.6)	9 (52.9)
Bad	1 (4.8)	1 (5.9)

Data are presented as n (%). ARIA: Allergic Rhinitis and its Impact on Asthma; GEMA: Spanish Asthma Management Guidelines. Step 5 corresponds to severe asthma requiring high-dose inhaled corticosteroids plus additional controller therapy. Percentages are calculated based on available data; missing data were not imputed. A small proportion of patients presented asthma without concomitant allergic rhinitis at baseline (asthma only), reflecting the real-world nature of the study population.

**Table 2:** Characteristics of local and systemic adverse reactions, n (%).

	Local Reactions n = 5 ^a^	Systemic Reactions n = 3 ^b^
Phase		
Up-dosing	4 (80.0)	1 (33.3)
Maintenance	1 (20.0)	2 (66.7)
Onset		
Immediate	3 (60.0)	1 (33.3)
Late	2 (40.0)	2 (66.7)
Intensity—Local reactions		
Mild	2 (60.0)	N/A
Moderate	2 (40.0)	N/A
Severe	0 (0)	N/A
Intensity—Systemic reactions (WAO criteria)		
Grade I	N/A	1 (33.3)
Grade II	N/A	2 (66.7)
Grade III–V	N/A	0 (0)
Serious adverse reactions	0 (0)	1 (33.3)
Treatments		
Local ice	5 (100)	0 (0)
Oral antihistamine	4 (80.0)	2 (66.7)
Topical corticosteroid	1 (20.0)	0 (0)
Corticosteroids IV/IM	0 (0)	1 (33.3)
Bronchodilators	0 (0)	1 (3.3)
Adrenaline	0 (0)	0 (0)
Other treatments ^c^	0 (0)	0 (0)

IM, intramuscular; IV, intravenous; N/A, not applicable; WAO, World Allergy Organization. ^a^ erythema + papular lesions + pruritus n = 2; erythema + papular lesion + pruritus + pain, n = 2; erythema + papular lesion, n = 1. ^b^ Bronchospasm, n = 2; unknown, n = 1. ^c^ Including intravenous antihistamine, oral corticosteroids, and fluid therapy, among others.

**Table 3:** Relationship between adverse reactions and sociodemographic, clinical, and treatment variables.

	Patients with AR n = 7	Patients with No AR n = 123	Total n = 130	*p*-Value
**Demographic variables**				
Age				0.100 ^b^
Children	0 (0)	17 (100)	17 (100)	
Adolescents	0 (0)	23 (100)	23 (100)	
Adults	7 (5.4)	83 (92.2)	90 (100)	
Sex				0.450 ^b^
Male	2 (1.5)	58 (44.6)	60 (46.2)	
Female	5 (3.8)	65 (50.0)	70 (53.8)	
**Clinical variables**				
Rhinitis diagnosis				0.586 ^a^
Yes	7 (5.4)	118 (90.8)	125 (96.2)	
No	0 (0)	5 (3.8)	5 (3.8)	
Asthma diagnosis				0.447 ^b^
Yes	5 (3.8)	64 (49.2)	69 (53.1)	
No	2 (1.5)	59 (45.4)	61 (46.9)	
**Treatment variables**				
Up-dosing schedule ^c^				0.463 ^b^
Cluster	6 (100.0)	106 (87.6)	112 (88.2)	
Conventional	0 (0)	15 (12.4)	15 (11.8)	

^a^ Chi square test with a 95% confidence level. ^b^ Unilateral Fisher test with a 95% confidence level. ^c^ Data for one patient was missing.

## Data Availability

The datasets generated and/or analyzed during the current study are not publicly available due to ethical, legal, and confidentiality restrictions related to clinical trial data and patient privacy, as well as sponsor confidentiality agreements, but are available from the corresponding author on reasonable request and with appropriate permissions.

## Supplementary Materials

The following supporting information can be downloaded at: https://www.mdpi.com/article/10.3390/diseases14020037/s1, Figure S1: Investigators’ opinion regarding the change in rhinitis, conjunctivitis, and asthma symptoms (A) and use of medication (B), and satisfaction with treatment (C), according to treatment (DP&DF vs. untreated); Figure S2: Patients’ opinion regarding the change in rhinitis, conjunctivitis, and asthma symptoms (A) and use of medication (B), according to treatment (DP&DF vs. untreated) and age (children, adolescents, and adults); Table S1: Demographic and clinical characteristics of study patients overall and according to age groups; Table S2: Patients’ sensitization profile based on skin prick test and specific IgE determinations (DP&DF group).

## Abbreviations

The following abbreviations are used in this manuscript:

AIT	Allergen immunotherapy
ARIA	Allergic Rhinitis and its Impact on Asthma
AR	Allergic rhinitis
ARs	Adverse reactions
DP&DF	*D. pteronyssinus*/*D. farinae*
GEMA	Guía Española para el Manejo del Asma
HDMs	House dust mites
LRs	Local reactions
SCIT	Subcutaneous immunotherapy
sIgE	Specific IgE
SRs	Systemic reactions
UT	Untreated
VAS	Visual analog scale
WAO	World Allergy Organization
