# Supplementary material for: Safety and Effectiveness of Subcutaneous Immunotherapy with a Glutaraldehyde-Polymerized Mite Allergen Extract in Adults and Children with Allergic Rhinitis with or Without Asthma Due to Dermatophagoides
Source: Diseases. 2026 Jan 23;14(2):37. doi: 10.3390/diseases14020037 (PMC12939221; doi:10.3390/diseases14020037)
Supplement: Supplementary file 1 [file diseases-14-00037-s001.zip › diseases-4082965-supplementary.pdf]

Supplementary Materials for the Submission "*Safety and Effectiveness of Subcutaneous Immunotherapy with a Glutaraldehyde-polymerized Mites Allergen Extract in Adults and Children with Allergic Rhinitis with or without Asthma due to Dermatophagoides*" by Verdeguer Segarra *et al*.

Supplementary Tables

Table S1. Demographic and clinical characteristics of study patients overall and according to age groups.

	Age groups			Total n=130
	**Children n=17**	**Adolescents n=23**	**Adults n=90**	
**Demographic characteristics**				
Sex, *n (%)*				
Male	9 (52.9)	13 (56.5)	38 (42.2)	60 (46.2)
Female	8 (47.1)	10 (43.5)	52 (57.8)	70 (53.8)
Age (years), *mean (SD)*	7.6 (2.1)	14.7 (1.5)	36.1 (11.0)	28.6 (14.7)
**Allergic disease characteristics, *n (%)***				
**Diagnoses**				
Rhinitis	16 (94.1)	23 (100)	86 (95.6)	125 (96.2)
Conjunctivitis	7 (41.2)	16 (69.6)	53 (58.9)	76 (58.5)
Asthma	15 (88.2)	7 (30.4)	47 (52.2)	69 (53.1)
**Rhinitis characteristics**	n=16	n=23	n=86	n=125
Frequency				
Intermittent	1 (6.3)	0 (0)	6 (7.0)	7 (5.6)
Persistent	15 (93.8)	23 (100)	80 (93.0)	118 (94.4)
Severity				
Mild	1 (6.3)	1 (4.3)	1 (1.2)	3 (2.4)
Moderate	10 (62.5)	19 (82.6)	63 (73.3)	92 (73.6)
Severe	5 (31.3)	3 (13.0)	22 (25.6)	30 (24.0)
Control				
Controlled	1 (6.3)	1 (4.3)	4 (4.7)	6 (4.8)
Partially controlled	5 (31.3)	14 (60.9)	33 (38.4)	52 (41.6)
Bad control	10 (62.5)	8 (34.8)	49 (57.0)	67 (53.6)
**Asthma characteristics**	n=14	n=7	n=45	n=66
Asthma treatment steps				
Step 1	3 (21.4)	2 (28.6)	6 (13.3)	11 (16.7)
Step 2	6 (42.9)	2 (28.6)	13 (28.9)	21 (31.8)

Step 3	3 (21.4)	2 (28.6)	16 (35.6)	21 (31.8)
Step 4	2 (14.3)	0 (0)	8 (17.8)	10 (15.2)
Step 5	0 (0)	0 (0)	1 (2.2)	1 (1.5)
Step 6	0 (0)	0 (0)	0 (0)	0 (0)
Not available	0 (0)	1 (14.3)	1 (2.2)	2 (3.0)
Classification				
Adults, n=45				
Intermittent	N/A	N/A	10 (22.2)	N/A
Persistent Mild	N/A	N/A	8 (17.8)	N/A
Persistent Moderate	N/A	N/A	25 (55.6)	N/A
Persistent Severe	N/A	N/A	2 (4.4)	N/A
Pediatric patients, n=21				
Occasional Episodic	4 (28.6)	3 (42.9)	N/A	N/A
Frequent Episodic	7 (50.0)	3 (42.9)	N/A	N/A
Persistent Moderate	3 (21.4)	1 (14.3)	N/A	N/A
Persistent Severe	0 (0)	0 (0)	N/A	N/A
Control				
Adults, n=45				
Well controlled	N/A	N/A	12 (26.7)	N/A
Partially controlled	N/A	N/A	22 (48.9)	N/A
Bad controlled	N/A	N/A	11 (24.4)	N/A
Pediatric patients, n=21				
Total	1 (7.1)	1 (14.3)	N/A	N/A
Good	5 (35.7)	3 (42.9)	N/A	N/A
Partial	7 (50.0)	3 (42.9)	N/A	N/A
Bad	1 (7.1)	0 (0)	N/A	N/A

ARIA, Allergic Rhinitis and its Impact on Asthma; GEMA, Guía Española para el Manejo del Asma; N/A, not applicable

Table S2. Patients' sensitization profile based on skin prick test and specific IgE determinations (DP&DF group).

	**DP&DF**<br>**n=130**	**Untreated**<br>**n=94**
**Skin prick test**		
Number of sensitizations, *mean (SD)/range*	3.61 (2.26)/0–12<br>n=125	3.61 (2.26)/0–9<br>n=85
Sensitizations, *n (%)*		
D. pteronyssinus + *D. farinae* + other allergens	71 (54.6)	22 (23.4)
D. pteronyssinus + *D. farinae*	49 (37.7)	0 (0)
Other allergens	2 (1.5)	48 (51.1)*
D. farinae + other allergens	1 (0.8)	0 (0)
D. pteronyssinus + other allergens	0 (0)	1 (1.1)
D. farinae	0 (0)	1 (1.1)
Not available	7 (5.4)	22 (23.4)
**Specific IgE**		
Number of sensitizations, *mean (SD)/range*	3.88 (1.75)/1–10<br>n=98	4.79 (2.62)/1–14<br>n=80
Sensitizations, *n (%)* n=130		
D. pteronyssinus + *D. farinae* + other allergens	61 (46.9)	21 (22.3)
Other allergens	17 (13.1)	51 (54.3)**
D. pteronyssinus + *D. farinae*	13 (10.0)	7 (7.5)
D. pteronyssinus	4 (3.1)	1 (1.1)
D. pteronyssinus + other allergens	3 (2.3)	0 (0)
Not available	32 (24.6)	14 (14.9)

SD, standard deviation

*Grass + *Olea europaea* + other allergens: n=30 (31.9%); Grass + *Olea europaea*: n=8 (8.5%); *Olea europaea* + other allergens: 8 (8.5%): *D. pteronyssinus* + *B. tropicalis* + other allergens: n=1 (1.1%); Grass + other allergens: n=1 (1.1%).
**Olea europaea* + other allergens: n=44 (46.8%); Grass + *Olea europaea* + other allergens: n=5 (5.3%); other allergens: 1 (1.1%); Grass + *Olea europaea*: n=1 (1.1%).

Supplementary Figures

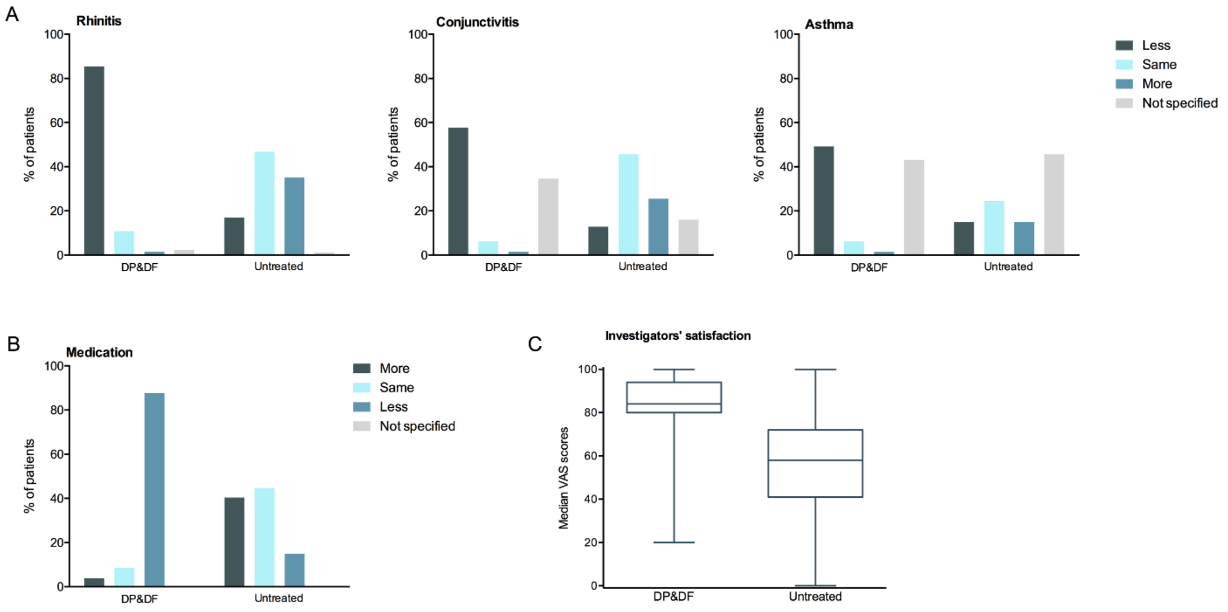

Figure S1. Investigators' opinion regarding the change in rhinitis, conjunctivitis, and asthma symptoms (A) and use of medication (B), and satisfaction with treatment (C), according to treatment (DP&DF vs untreated). AIT, allergen immunotherapy. In (C), the box represents the interquartile range (Q1, Q3), the line in the middle of the box represents the median, and the whiskers represent the minimum and maximum.

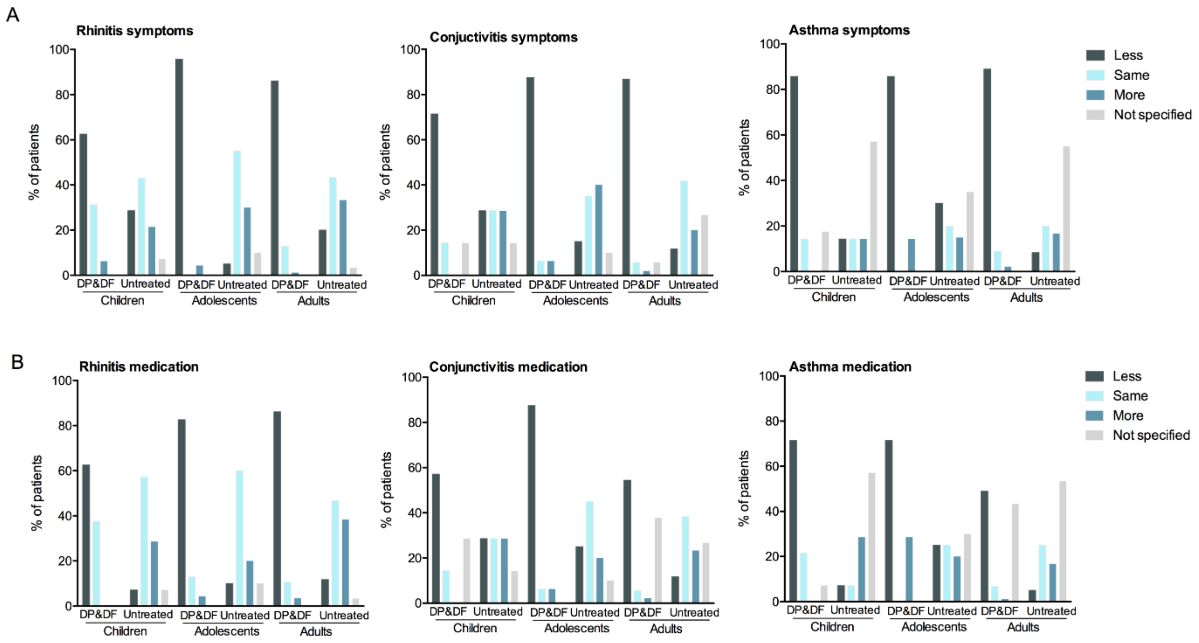

Figure S2. Patients' opinion regarding the change in rhinitis, conjunctivitis, and asthma symptoms (A) and use of medication (B), according to treatment (DP&DF vs untreated) and age (children, adolescents, and adults). AIT, allergen immunotherapy.
